# Molecular genetic bases of seed resistance to oxidative stress during storage

**DOI:** 10.18699/VJ20.47-o

**Published:** 2020-08

**Authors:** N.А. Shvachko, E.K. Khlestkina

**Affiliations:** Federal Research Center the N.I. Vavilov All-Russian Institute of Plant Genetic Resources (VIR), St. Petersburg, Russia; Federal Research Center the N.I. Vavilov All-Russian Institute of Plant Genetic Resources (VIR), St. Petersburg, Russia

**Keywords:** seeds, barley, QТL, seed longevity genes, genetic markers, biochemical markers, семена, ячмень, QТL, гены долголетия семян, генетические маркеры, биохимические маркеры

## Abstract

Conservation of plant genetic diversity, including economically important crops, is the foundation for food safety. About 90 % of the world’s crop genetic diversity is stored as seeds in genebanks. During storage seeds suffer physiological stress consequences, one of which is the accumulation of free radicals, primarily reactive oxygen species (ROS). An increase in ROS leads to oxidative stress, which negatively affects the quality of seeds and can lead to a complete loss of their viability. The review summarizes data on biochemical processes that affect seed longevity. The data on the destructive effect of free radicals towards plant cell macromolecules are analyzed, and the ways to eliminate excessive ROS in plants, the most important of which is the glutathioneascorbate pathway, are discussed. The relationship between seed dormancy and seed longevity is examined. Studying seeds of different plant species revealed a negative correlation between seed dormancy and longevity, while various authors who researched Arabidopsis seeds reported both positive and negative correlations between dormancy and seed longevity. A negative correlation between seed dormancy and viability probably means that seeds are able to adapt to changing environmental conditions. This review provides a summary of Arabidopsis genes associated with seed viability. By now, a significant number of loci and genes affecting seed longevity have been identified. This review contains a synopsis of modern studies on the viability of barley seeds. QTLs associated with barley seed longevity were identified on chromosomes 2H, 5H and 7H. In the QTL regions studied, the Zeo1, Ale, nud, nadp-me, and HvGR genes were identified. However, there is still no definite answer as to which genes would serve as markers of seed viability in a certain plant species.

## Abstract (ru)

Сохранение генетического разнообразия растений, в том числе хозяйственно значимых культур, является основой продовольственной безопасности. В мире около 90 % генетического разнообразия культурных растений сохраняется в виде семян в генных банках. В процессе хранения в семенах накапливаются свободные радикалы, в первую очередь активные формы кислорода (АФК). Повышение уровня АФК вызывает окислительный стресс, который негативно влияет на качество семян и может при- вести к полной потере их жизнеспособности. В обзоре обобщены сведения о биохимических процессах, влияющих на продолжительность жизни семян. Проанализированы данные о деструктивном действии свободных радикалов по отношению к макромолекулам клетки растения и пути устранения избыточного количества АФК в растениях, наиболее важным из которых является аскорбат-глутатионовый путь. Рас- сматривается вопрос взаимосвязи периода покоя и длительности сохранения семян. В исследованиях на семенах разных видов растений была выявлена отрицательная корреляция между периодом покоя и долголетием семян, тогда как в работах с семенами Arabidopsis различные авторы выявили как положи- тельную корреляцию между периодом покоя и длительностью сохранения семян, так и отрицательную. Отрицательная корреляция между периодом покоя и жизнеспособностью, вероятно, свидетельствует о способности семян адаптироваться к изменяющимся условиям окружающей среды. Нами проанализи- рована информация по генам Arabidopsis, связанным с жизнеспособностью семян. В настоящее время выделено значительное количество локусов и генов, влияющих на долголетие семян. Статья знакомит с современными исследованиями жизнеспособности семян ячменя. Локусы количественных призна- ков (QTL), связанные с долголетием семян ячменя, были определены на хромосомах 2Н, 5Н и 7Н. В изученных областях QTL выявлены гены Zeo1, Ale, nud, nadp-me и HvGR. Однако вопрос о том, какие гены являются маркерами жизнеспособности семян растений определенного вида, остается открытым.

## Introduction

Conservation of plant genetic diversity, including economically important crops, is a task of topmost priority. Since the end of the previous century plant genebanks have been sprouting up all over the world. Currently, about 90 % of crop accessions are stored worldwide in genebanks as seeds (Li, Pritchard, 2009; http://www.fao.org). The N.I. Vavilov Institute of Plant Genetic Resources (VIR) holds over 320,000 viable accessions of cultivated plants and their wild relatives, including more than 250,000 seed accessions in its Kuban Seed Genebank, founded in 1976 (Loskutov, 2009; Silaeva, 2012).

Seeds represent a stage in the life cycle when plants suffer particularly high levels of genotoxic stress, which can lead to the genome’s instability (Waterworth et al., 2011). Seed ageing is regarded as the accumulation of structural and metabolic injuries leading to functional failures and reduced resistance to unfavorable environments, which may result in loss of viability (McDonald, 1999; Smolikova, 2014). According to their behavior under storage, seeds are classified into orthodox and recalcitrant (Walters, 2015). In the end of their ripening period, orthodox seeds, as a rule, lose water and dry out down to a moisture content of 10 %; they can be preserved in such state for many years without any loss of germination rate. This property of orthodox seeds was called ‘desiccation tolerance’ (Dekkers et al., 2015). Contrariwise, dehydration in recalcitrant seeds entails loss of their germination ability and death. Therefore, they are considered desiccation-sensitive. Usually recalcitrant seeds are not dried prior to their placement in a genebank for conservation. Orthodox seeds are dried down to a moisture content of 5 % or less, or frozen before storage. As a rule, the duration of storage periods for orthodox seeds in genebanks may be extended if they are kept under lower moisture and temperature conditions (Bewley et al., 2013). For example, barley seeds retain their viability from six months to 7–9 years at the air temperature of +20 °C and relative humidity (RH) of 50 % (Priestley et al., 1985; Nagel, Börner, 2010), whereas under negative temperatures (‒18 °C) and 4–8 % RH, according to calculated estimates, seed viability may remain intact up to more than 80 years (Walters et al., 2005).

In addition to environmental factors, such as humidity, temperature, light, and absence of pathogens (Schmidt, 2000), seed ageing rates are affected by genetic factors determining seed color and weight and influencing the activity of protein non-enzymatic glycosylation processes, lipid peroxidation, cell membrane structure, generation and neutralization of reactive oxygen species (ROS) and free radicals, and other processes (Wettlaufer, Leopold, 1991; Ponquett et al., 1992; Khan et al., 1996; Wojtyla et al., 2016; Frolov et al., 2018; Antonova et al., 2019).

The causes of seed viability losses and deaths are not quite clear, because a lot of processes are involved, including damage to macromolecules (such as DNA, lipids and proteins) resulted from ROS-induced responses. This issue was studied by numerous researchers. Using various plant species as examples, they showed that seed ageing rates depended on protection mechanisms against stress and the ability of seeds to withstand ROS-induced changes. In different plant species antioxidants are differently involved in removing excessive ROS. In oil crops, for example, a more active role is played by lipophilic antioxidants (Bailly, 2004; Bahin et al., 2011; Waterworth et al., 2011; Jeevan Kumar et al., 2015; Kong et al., 2015). The research efforts employing germplasm materials preserved in the IPK (Germany) and the USDA (United States) genebanks showed that seed longevity varied not only across different plant species, but also among different genotypes within a single species, although interspecific differences in seed shelf-life exceeded intraspecific ones. Similar research was performed on different plant species: barley, wheat, rapeseed, etc. (Walters et al., 2005; Nagel et al., 2009; Nagel, Börner, 2010; Rehman Arif et al., 2017; Rehman Arif, Börner, 2019). The fact that genotypes within the same species demonstrated different seed life spans may serve as a basis for genetic analysis of seed longevity.

## Seed dormancy and longevity

Physiological dormancy may be described as a preprogrammed state limiting the set of environmental conditions under which the seed germinates (Nikolaeva, 1982, 1999; Baskin J., Baskin C., 2007). Dormancy is an adaptive trait that enables seeds to survive lengthy periods of unfavorable conditions (Bewley et al., 2013; Sliwinska, Bewley, 2014). To optimize germination and maintain viability during long unfavorable periods, seeds enter into the state of dormancy (Rajjou, Debeaujon, 2008). Seed dormancy is regulated through abscisic acid (ABA) and other bioactive compounds, such as plant hormones: gibberellins, cytokinins, and ethylene (Nonogaki, 2017). Entering the state of seed dormancy is accompanied by a considerable increase in ABA content, whereas releasing from dormancy, on the contrary, is associated with a decrease in ABA. 

The issue of the interplay between seed dormancy and seed longevity has been analyzed by numerous researchers for decades, although the conclusions made on the results of those studies are contradictory. Clerkx et al. (2004) studied this problem on Arabidopsis plants with mutations in certain developmental and biochemical pathways. They reported reduced seed longevity for such mutations as abscisic acid insensitive3 (abi3) and abscisic acid deficient1 (aba1). The same mutations also provoked reduction of seed dormancy duration for abi3 and aba1. Thus, the authors supposed that a positive correlation existed between seed longevity and dormancy. Miura et al. (2002), while studying interrelations between dormancy and seed viability in rice, came to an opposite conclusion – that these two physiological states are controlled by different genetic factors. The QTL analysis showed that the seed longevity loci were on chromosomes 2, 4 and 9, while the QTLs associated with seed dormancy were identified on chromosomes 1, 3, 5, 7 and 11. Nguyen et al. (2012) also observed a negative correlation between seed longevity and dormancy in Arabidopsis plants. Having analyzed six populations of recombinant inbred lines (RILs), the authors identified 5 loci linked with seed germination ability after storage (GAAS1–GAAS5). They reported that the GAAS (germination ability after storage) loci correlated with those responsible for seed dormancy, i. e. DOG (delay of germination) loci. Their correlation was negative. A detailed analysis of GAAS5 and DOG1 QTLs revealed that the DOG1 locus reduces seed longevity and at the same time increases seed dormancy period (Nguyen et al., 2012). A homolog of the same gene was annotated by Nagel et al. (2019) on the barley chromosome 3H. The authors reported that the DOG1 locus plays a role in extending the period of barley seed dormancy, although a less significant role than in the case of Arabidopsis.

Thus, the interaction between seed dormancy and seed longevity is interpreted ambiguously, so further research would be required to clarify this issue. The presence of loci that either extend the life span of seeds or prolong their dormancy period within an individual plant accession may provide it with adaptive plasticity, which will lead to the manifestation of an optimal phenotype under various environmental conditions.

## Biochemical processes affecting seed longevity

In the process of storage, seeds suffer physiological stress consequences, one of which is the accumulation of free radicals, primarily reactive oxygen species (ROS). An increase in the ROS level results in oxidative stress. In most cases under oxidative stress a superoxide anion radical (superoxide radical, O2·) is formed; it quickly converts into other ROS forms: hydrogen peroxide (H2O2) and the hydroxyl radical (· OH). Damage caused by free radicals leads to a rupture in the genome integrity within the nucleus, which in its turn may entail a complete loss of seed viability (Bailly, 2004; Kranner et al., 2006; Bailly, Kranner, 2011). The effect of free radicals and lipid peroxidation induces membrane structure degradation and DNA disintegration, accompanied by a decrease in the activity of a majority of enzymes in the cell. One of the causes of such decline in activity may be the deterioration of enzymes or malfunctions in the protein-synthesizing complex under the aggregate impact of free radicals. There is a universal mechanism of oxidative stress prevention, usable against excessive ROS in plants. An important role in the antioxidant protection system of the cell is played by the enzymes of superoxide dismutase (SOD). The main function of these enzymes is converting the superoxide radical into H_2_O_2_ and oxygen (Jajic et al., 2015). Three SOD isoforms differing in metals within the active center have been identified in plant cells: Fe-SOD (chloroplast), Mn-SOD (mitochondria and peroxisomes) and Cu-Zn-SOD (cytoplasm, chloroplast and peroxisomes). Hydrogen peroxide is in its turn decomposed by catalase (CAT), which is found in glyoxysomes and peroxisomes (Willekens et al., 1995), except the isoform which is mitochondrial (Scandalios et al., 1997). Glutathione peroxidase (GPX) also removes excessive Н_2_O_2_ and organic peroxides (Eshdat et al., 1997).

The ascorbate-glutathione or the Foyer–Halliwell–Asada pathway also plays a significant role in the cell’s antioxidant protection (Bailly, 2004; Foyer, Noctor, 2005) (see the Figure). In this pathway, Н_2_O_2_ reduction to water is catalyzed by ascorbate peroxidase. It is accompanied by ascorbate oxidation to monodehydroascorbate (MDA), which can be reduced back to ascorbate by MDA reductase, employing NADPH. As a result of a spontaneous redox reaction, MDA can produce dehydroascorbate (DHA), which is converted back to ascorbate via glutathione oxidation. The cycle is completed with the NADPH-assisted reduction of the oxidized glutathione form (Medvedev, 2013).

**Fig. 1.**
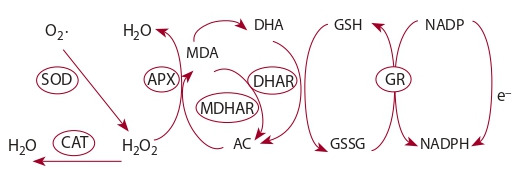
Ascorbate-glutathione pathway: CAT – catalase; SOD – superoxide dismutase; APX – ascorbate peroxidase; МDHАR – monodehydroascorbate reductase; DHAR – dehydroascorbate reductase; GR – glutathione reductase; AC – ascorbate; MDA – monodehydroascorbate; DHA – dehydroascorbate; GSSG – oxidized glutathione; GSH – reduced glutathione.

Redox potential of the water-soluble antioxidant glutathione (GSH) is of particular importance as a regulator of the cell’s redox potential in the case of orthodox seeds (Schafer, Buettner, 2001; Noctor et al., 2011). Since orthodox seeds contain insignificant amounts of ascorbate (Kranner et al., 2006), Kranner and her coauthors hypothesized that glutathione is the main and possibly the oldest redox buffer, and a change in the GSSG/GSH reduction potential can serve as a universal seed viability marker (Mittler, 2002; Kranner et al., 2006). In addition to watersoluble antioxidants, plant seeds also contain fat-soluble (hydrophobic) antioxidants: α-, β-, γ-, σ-tocopherols and carotenoids (Sharova, 2016); their contribution to the cell’s antioxidant protection depends on the plant species. For example, fat-soluble antioxidants are more significant for oil crops whose seeds are rich in fatty acids.

Seed longevity is also affected by some other compounds with an antioxidant function, such as polyphenols, flavonoids and peroxiredoxins (Landry et al., 1995; Sattler et al., 2004; Sharova, 2016). It should be mentioned that the cell’s antioxidant mechanisms control the content of ROS, but do not eliminate them completely. It happens because small amounts of ROS are important signaling molecules, participating in plant growth, development, and stress response. In seeds, ROS play an important role, associated with viability and release from dormancy. An excess in ROS leads to a loss of seed viability, but ROS production is required for seed dormancy breakage and facilitates seed germination (Bailly, 2004; Oracz et al., 2009).

Thus, ROS play a dual role in seed physiology. On the one hand, ROS possess an exceptionally high reactivity. They are capable to induce chain reactions and oxidize practically all organic compounds, causing irreversible oxidative damage to most important biomolecules, such as proteins, lipids or DNA. On the other hand, ROS participate in cell growth regulation, protection against pathogens, and redox status control in cells. Besides, ROS function as a positive signal in seed dormancy alleviation and germination (Bahin et al., 2011; Jeevan Kumar et al., 2015).

## Candidate genes for seed longevity identified in Arabidopsis thaliana (L.)

The role of important factors affecting seed longevity was analyzed in mutants of Arabidopsis thaliana (L.) Heynh. and transgenic lines. Seed maturation is known to be genetically controlled by four main regulators: ABI3 (ABSCISIC ACID-INSENSITIVE3), LEC1, LEC2 (LEAFY COTYLEDON1 and LEAFY COTYLEDON2) and FUS3 (FUSCA3) (Raz et al., 2001). Mutations in these key regulators lead to a quick loss of seed viability under storage. For example, аbi3, lec1 and fus3 mutants demonstrated reduced seed longevity (Ooms et al., 1993; Clerkx et al., 2004). Significant reduction of seed longevity is also caused by seed coat mutations. Seed coat acts as a structural barrier against biotic and abiotic stresses. For example, tt (transparent testa), ttg (transparent testa glabra) and ats (aberrant testa shape) mutants were reduced germination percentage than the control plants (Debeaujon et al., 2000; Clerkx et al., 2004). A decrease of seed longevity was observed in vte1 and vte2 mutants involved in vitamin E biosynthesis (lipophilic antioxidant) and in completely tocopherol-free ones (Sattler et al., 2004).

After UV exposure, Arabidopsis mutants deficient in flavonoid biosynthesis showed increased lipid peroxidation by 60 %, which correlated with a decrease in seed viability (Landry et al., 1995). The content of some oligosaccharides, such as galactinol, correlated with seed longevity (Obendorf, 1997). The research conducted on Arabidopsis, cabbage and tomato revealed a positive correlation between galactinol content and seed longevity in these crops (de Souza Vidigal et al., 2016). Reduced seed viability, compared with the wild-type plants, was observed in Arabidopsis mutants that lacked the functional malate hydrogenase enzyme (Yazdanpanah et al., 2019). The researchers who made such observation assume that the NADP-ME1 enzyme activity is required to protect seeds from oxidation during long-term seed storage (Yazdanpanah et al., 2019). Oxidative stress negatively affects seed quality; it is confirmed by a reduction in germination percentage of the frostbite1 ( fro1) mutant which constitutively accumulated ROS (Lee et al., 2002; Clerkx et al., 2004). At the same time, Clerkx et al. failed to find a reliable reduction of germination rate for two mutants with ROS-scavenging mechanisms: vitamin C deficient1-1 (vtc1-1) and cadmium sensitive2-1 (cad2-1), the latter being glutathione-deficient.

By now, a considerable number of loci and genes associated with seed longevity have been identified in Arabidopsis plants. Such genes may be classified into several groups according to the mechanisms of their effect on seed viability; the most significant among them are the groups of genes whose effect is connected with plant hormones, such as abscisic and gibberellic acids, and redox processes.

## Candidate genes for seed longevity identified in barley

Studying seed longevity of various plant species, such as Arabidopsis (Clerkx et al., 2004; Bentsink et al., 2006), barley (Nagel et al., 2009), wheat (Landjeva et al., 2010; Rehman Arif, Börner, 2019), rice (Miura et al., 2002), Aegilops (Landjeva et al., 2010), maize (Revilla et al., 2009), lettuce (Schwember, Bradford, 2010), etc., has shown that seed longevity is controlled by several genetic factors, which facilitates the identification of quantitative trait loci (QTLs). In a number of published works QTLs associated with seed longevity were identified for different plant species using natural and artificial ageing techniques. Artificial ageing methods are employed to predict eligibility of seed samples for long-term storage (Safina, Filipenko, 2013; Smolikova, 2014). They are essentially based on artificial acceleration of the senescence process by briefly exposing seeds to higher temperatures and humidity close to critical levels for a given crop. There are two generally accepted seed ageing methods: AA (accelerated ageing) and CD (controlled deterioration). In the AA test, seeds are exposed for short period of time to high temperature and high relative humidity (100 %). CD treatment differs from AA in that seeds are shortly stored under higher moisture content (MC) (18–20 %) and temperatures in aluminum foil bags (Nagel et al., 2009, 2015). The ISTA (International Seed Testing Association) standards prescribe a definite MC value to the CD test according to the following formula:

**Formula:**
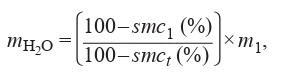
1

where mH_2_O is the amount of added water (g); smc_1_ is the initial seed moisture content (%); smc_t_ is the taget seed moisture content (%); and m_1_ is the initial seed weight (g).

It should be mentioned that under accelerated ageing seeds are experiencing high water contents and high temperatures. In such a situation, the main negative processes within the seeds are lipid peroxidation and loss of membrane phospholipids. Meanwhile, during long-term storage of dry seeds, mostly non-enzymatic reactions would take place, and they do not require much water (Walters, 1998; Murthy et al., 2003; Veselovsky, Veselova, 2012; Frolov et al., 2018; Antonova et al., 2019). Therefore, the question on the extent to which the processes within seeds under artificial ageing would mimic those undergoing naturally during long-term storage remains unanswered (Agacka- Mołdoch et al., 2016; Bankin et al., 2018). Nonetheless, artificial ageing techniques are widely used by various researchers in their efforts to study seed viability.

The development of new sequencing methods, achievements in a wide range of analytical technologies, and emergence of bioinformatics procedures contributed to higher quality of research not only on model species but also on agricultural crops. Several significant specific features of Hordeum vulgare (L.), such as the diploid nature of the cultivated barley genome with a high degree of self-pollination, small number of sufficiently large chromosomes (2n = 14), easy crossability, and simplicity of cultivation in very diverse climates, promote the wide use of this crop in genetic studies. Modern DNA technologies applied to various barley accessions helped to identify candidate genes potentially responsible for extending the life span of seeds. Nagel et al. in 2009 used two induced ageing methods (AA and CD) to detect QTLs correlating with seed longevity in the following barley plant populations: OWB, S × M (‘Steptoe’ × ‘Morex’) and W766 (Nagel et al., 2009). The authors identified the greatest number of QTLs associated with seed longevity on chromosomes 2H, 5H and 7H. One significant QTL was detected within a distal region of chromosome 2H associated with the gene Zeo1 (Zeocriton 1). The Zeo1 gene determines plant height and spike compactness. Such plants are known to have low fertility. The Ale (Aleurain) gene encoding thiol protease was identified in the QTL on the long arm of chromosome 5H. The expression of Ale is regulated by gibberellic and abscisic acids which play important roles in seed germination (Nagel et al., 2009). The nud gene determining hulled/naked caryopsis was identified as a candidate for QTL on chromosome 7H. In 2016, Nagel et al. conducted additional research on seed longevity. Most of the QTLs associated with seed longevity, like in the 2009 tests, were detected in two areas: on chromosome 2H between 110 and 172 cM, the locality of the Zeo1 gene, and in the centromeric region of 7H from 73 to 95 cM, also incorporating the nud gene.

Further annotation of gene functions in the QTL areas by the authors revealed the presence of the glutathione reductase enzyme in the same area, which suggests a relationship with oxidative stress (Meyer, Hell, 2005; Rouhier et al., 2008; Nagel et al., 2016). Bahin et al. studied sunflower and barley seed longevity in 2011 and found out that in the process of storage barley seeds, unlike those of sunflower, did not accumulate ROS. The authors supposed that an excess in ROS neutralized the antioxidant glutathione (GSH) (Bahin et al., 2011), which could serve as a marker in barley seed longevity studies. Wozny et al. in 2018 proposed the NADP-dependent malic enzyme (NADP-ME) as a candidate gene to be used in future seed longevity studies on barley (Wozny et al., 2018). The enzyme was localized on chromosome 2H within the area of the QTLs correlating with seed longevity in the studied barley accessions (Wozny et al., 2018).

Thus, most of the QTLs associated with barley seed longevity have been identified on chromosomes 2Н, 5Н and 7Н. The following genes have been detected in the studied QTL areas: Zeo1, Alе, nud, nadp-me, and HvGR. Different researchers offer different candidate genes for further research on barley seed longevity. By now, however, no one has found a reliable universal marker to be associated with loss of viability in barley seeds. Presently, there are no readily available genetic markers for seed longevity that could prove handy to search for an optimum frequency of seed reproduction in the process of barley germplasm conservation.

## Conclusion

Mechanisms of seed viability reduction and death during long-term storage may differ depending on plant species. For barley, they have been identified only partially. The data presently available on the identification of genes associated with barley seed longevity have been obtained using artificial seed ageing methods, but they cannot simulate natural ageing processes accurately enough. Genetic markers associated with seed longevity that could appear suitable to optimize reproduction of seed accessions have not yet been conclusively identified. Eventually, glutathione reductase may be suggested as a candidate gene for further studies of barley seed longevity. This enzyme is directly involved in neutralization of excessive ROS, antioxidant defense, and cell signaling in plants.

## Conflict of interest

The authors declare no conflict of interest.
